# Differential Diagnostic Value of Histology in MPLC and IPM: A Systematic Review and Meta-Analysis

**DOI:** 10.3389/fonc.2022.871827

**Published:** 2022-04-29

**Authors:** Sen Tian, Fuqi Li, Jin Pu, Yi Zheng, Hui Shi, Yuchao Dong, Ruohua Chen, Chong Bai

**Affiliations:** ^1^ Department of Respiratory and Critical Care Medicine, Shanghai Changhai Hospital, the First Affiliated Hospital of Second Military Medical University, Shanghai, China; ^2^ Department of Pathology, Shanghai Changhai Hospital, the First Affiliated Hospital of Second Military Medical University, Shanghai, China; ^3^ Department of Special Diagnosis and Treatment, Shanghai Changhai Hospital, the First Affiliated Hospital of Second Military Medical University, Shanghai, China; ^4^ Department of Statistics, Shanghai Changhai Hospital, the First Affiliated Hospital of Second Military Medical University, Shanghai, China

**Keywords:** multiple primary lung cancer, intrapulmonary metastasis, histology, meta-analysis, molecular

## Abstract

**Background:**

The paramount issue regarding multiple lung cancer (MLC) is whether it represents multiple primary lung cancer (MPLC) or intrapulmonary metastasis (IPM), as this directly affects both accurate staging and subsequent clinical management. As a classic method, histology has been widely utilized in clinical practice. However, studies examining the clinical value of histology in MLC have yielded inconsistent results; thus, this remains to be evaluated. Here, we performed a meta-analysis to assess the differential diagnostic value of histology in MPLC and IPM and to provide evidence-based medicine for clinical work.

**Methods:**

PubMed, Embase, and Web of Science databases were searched to collect relevant literature according to PRISMA, and inclusion and exclusion criteria were set up to screen and assess the literature. The data required for reconstructing a 2 × 2 contingency table were extracted directly or calculated indirectly from the included studies, and statistical analysis was carried out by using Stata 15, Meta-DiSc 1.4, and Review Manager 5.4 software.

**Results:**

A total of 34 studies including 1,075 pairs of tumors were included in this meta-analysis. Among these studies, 11 were about the M-M standard and the pooled sensitivity and specificity were 0.78 (95% CI: 0.71–0.84) and 0.47 (95% CI: 0.38–0.55), respectively; 20 studies were about CHA and the pooled sensitivity and specificity were 0.76 (95% CI: 0.72–0.80) and 0.74 (95% CI: 0.68–0.79), respectively; and 3 studies were about the “CHA & Lepidic” criteria and the pooled sensitivity and specificity were 0.96 (95% CI: 0.85–0.99) and 0.47 (95% CI: 0.21–0.73), respectively. The combined pooled sensitivity, specificity, PLR, NLR, DOR, and the area under the SROC curve of the 34 studies were 0.80 (95% CI: 0.73–0.86), 0.64 (95% CI: 0.51–0.76), 2.25 (95% CI: 1.59–3.17), 0.31 (95% CI: 0.23–0.43), 7.22 (95% CI: 4.06–12.81), and 0.81 (95% CI: 0.77–0.84), respectively.

**Conclusion:**

The current evidence indicated that histology had a moderate differential diagnostic value between MPLC and IPM. Among the three subgroups, the “CHA & Lepidic” criteria showed the highest sensitivity and CHA showed the highest specificity. Further research is necessary to validate these findings and to improve clinical credibility.

**Systematic Review Registration:**

PROSPERO, identifier CRD42022298180.

## Introduction

There has been an increasing number of multiple lung cancer (MLC) patients that are being diagnosed due to advances in high-resolution computed tomography (HRCT) and increased awareness among clinicians regarding MLC screening. Recent reports have shown that the incidence rate of MLC ranges from 2.4% to 18.7% (1–6). Hence, an accurate discrimination of multiple primary lung cancer (MPLC) and intrapulmonary metastasis (IPM) is of great clinical significance since this may assist in TNM classification and optimizing therapeutic options (1, 3, 4, 6–10). In the eighth edition of the TNM classification of lung cancer, multiple nodules within the same lobe are categorized as T3, different but ipsilateral lobes as T4, and contralateral lobes as M1. However, this staging is based on the supposition that nodules are IPM. Therefore, the TNM staging system is significantly excessive for patients with MPLC, thus hindering the administration of surgical resection with curative intent and offering palliative therapy to MPLC patients.

In 1975, Martini and Melamed (11) initially proposed the criteria to diagnose MPLC based on tumor locations and histological characteristics, which remained the primary method in the clinical field since the mid-1970s, thanks to the simple and operable benefit of the standard itself. However, the criteria are rather empirical and have proven to be difficult when the histological features are similar. The existence of intratumor heterogeneity (12) sheds light on determining whether MLC is MPLC or IPM. In detail, the histological variation manifests a significant diversity of structural and cytological characteristics in an individual tumor, accompanied by the variation of stromal characteristics and related inflammatory environment, which endows tumors with distinctive histologic characteristics. In view of this, a landmark study has demonstrated that comprehensive histologic assessment (CHA) (i.e., percentages of the histologic subtypes and distinctive histologic characteristics such as degree of keratinization, amount of necrosis, and quality of stroma including the pattern of desmoplasia or inflammation) could be utilized to accurately differentiate MPLC from IPM (13) and had gained great popularity in the clinical setting. More recently, Sun et al. (5) proposed that the “CHA & Lepidic” criteria (i.e., CHA combined with a low-grade lepidic component) distinguished between MPLC and IPM. The rationale is that a lepidic component is the most significant characteristic of atypical adenomatous hyperplasia (AAH) and adenocarcinoma *in situ* (AIS), and MLC patients with a lepidic component have a better prognosis outcome (14). The “CHA & Lepidic” standard showcases a promising method of the accurate and cost-effective distinction of MPLC from IPM. Clearly, further research is necessary to fully assess the clinical value of the “CHA & Lepidic” criteria.

In the past few decades, various kinds of powerful and refined methods of molecular biology, such as array-based comparative genomic hybridization (aCGH) (1, 5, 13, 15), next-generation sequencing (NGS) (3, 4, 6, 8–10, 16–26), expression of proteins (7), microsatellite instability (MSI) (27, 28), and miRNA (29), have been used in the discrimination of MPLC and IPM. As the most accurate method of differentiating MPLC from IPM, molecular analysis can not only exhibit marked differences in biologic behavior but also yield individualized and forecasting therapeutic options for patients. Nevertheless, the method is commonly utilized by scientific research or auxiliary diagnosis and, regretfully, cannot be adopted in routine clinical practice due to its high requirements for technology, equipment, and economic circumstances. Histology remains in mainstream use in the clinical field at any given moment. It has to be mentioned that inconsistencies exist among studies that have examined the clinical value of histology in MLC. In addition, the differential diagnostic value of histology between MPLC and IPM was evaluated in the subtype of M-M standard, CHA, and “CHA & Lepidic” criteria separately, never combined nor systematically compared. Herein, to our knowledge, we performed the first and most comprehensive meta-analysis of all eligible studies that used the highly robust molecular analysis as the gold standard to assess the differential diagnostic value of histology in MPLC and IPM and to compare the diagnostic performance of the M-M standard, CHA, and “CHA & Lepidic” criteria.

## Methods

### Search Strategy

We comprehensively searched relevant articles using PubMed, Embase, and Web of Science databases from 1 January 2000, to 1 September 2021. The terms “synchronous,” “separate,” “multifocal,” “multiple primary lung cancer,” “molecular,” “genomic,” “next-generation sequencing,” and “Lung Neoplasms,” either as keywords or as Medical Subject Headings (MeSH) terms, were searched in different combinations. Two investigators (FL, JP) performed the search strategy independently and then conducted a secondary retrieval of eligible studies. Apart from database retrieval, the reference list of eligible literature was also manually screened to identify potentially relevant studies not included in the initial search. The detailed search strategy is listed in the **Supplementary Table**.

### Selection Criteria

Two independent researchers (FL, JP) assessed potentially relevant articles, according to the following selection criteria, and the discrepancies were checked by performing a blind cross-check. If there were any disagreements, the inconsistencies were solved by another reviewer (HS).

Eligible studies must meet the following criteria: a) human-based studies; b) the definitions of MPLC and IPM criteria should be explicitly explained; c) there should be at least 10 pairs of tumors within each study; d) relevant data of reconstructing a 2 × 2 table [i.e., true positive (TP), false positive (FP), true negative (TN), and false negative (FN)] were extracted directly or calculated indirectly from the included literature; and e) the study included both molecular analysis and histology as the differential diagnostic methods of MPLC and IPM, and molecular analysis was the gold standard in each of the studies.

The exclusion criteria were as follows: a) reviews, conference abstracts, case reports, editorials, guidelines, comments, or letters to the editor; b) language not in English; c) articles with low quality based on QUADAS-2 guidelines; and d) unavailable or incomplete data to reconstruct a 2 × 2 contingency table.

### Data Extraction and Quality Assessment

Two independent reviewers (ST, FL) investigated all eligible articles and extracted the following information in a standardized form: first author, year of publication, country, cancer type, the number of tumor pairs, histological method, TP, FP, TN, FN, sensitivity, specificity, and consistency. If some essential data were needed, the corresponding authors would be contacted. Afterward, two researchers independently performed the quality assessment and any inconsistencies were adjudicated by a third investigator (YZ). The QUADAS-2 checklist was applied to assess the quality of the included studies.

### Statistical Analysis

A 2 × 2 contingency table was tabulated to sort the date, including information regarding TP, FP, TN, and FN. The threshold effect and non-threshold effect were used to evaluate the heterogeneity of the included studies. A Spearman rank correlation was adopted to estimate whether the heterogeneity was caused by the threshold effect. Cochran’s *Q* test, Higgins’ *I* ^2^ test, and forest plots were used to confirm if the heterogeneity originated from the non-threshold effect; *I* ^2^ values between 0% and 24%, 25% and 49%, 50% and 74%, and greater than 75% implied no, low, medium, and high heterogeneity separately (30). If a non-threshold effect existed, the random-effects model approach would be performed in this study. Meta-regression analysis and subgroup analysis were utilized to explore the source of potential heterogeneity. A funnel plot was used to investigate publication bias. Stata 15, Meta-DiSc 1.4 (31), and Review Manager 5.4 software were employed to perform the statistical analysis. *P*-values less than 0.05 indicated statistical significance.

## Results

### Literature Search

**Figure 1:**
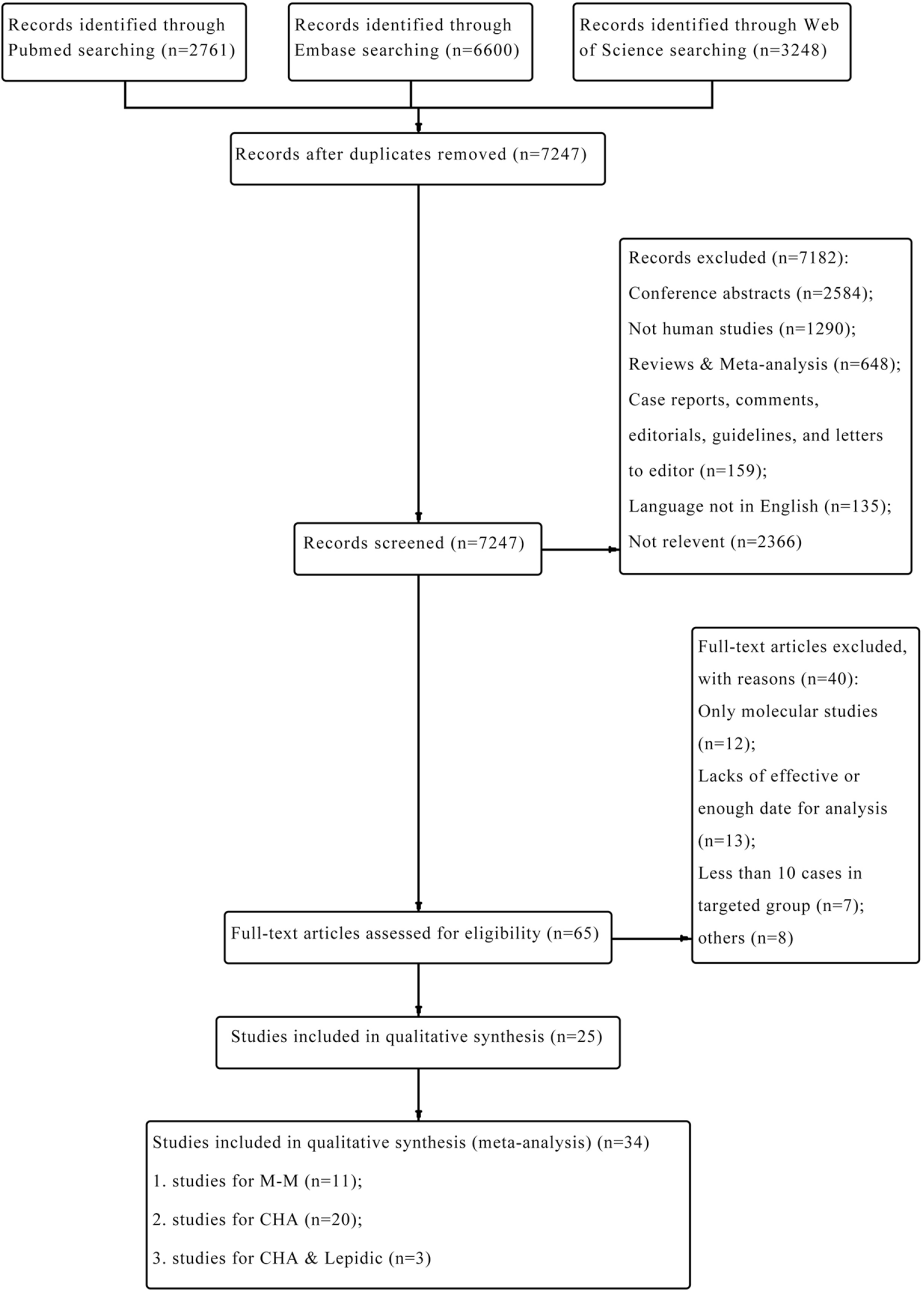
The PRISMA flow diagram of the selected eligible studies.

A total of 12,609 potentially relative studies were retrieved from the three databases. As presented in **Figure 1**, after screening, 65 articles were assessed for eligibility and 25 articles were included. According to different histological methods, 34 studies including 1,075 pairs of tumors were finally retrieved in this meta-analysis. Among these studies, 11 reported the diagnostic performance of the M-M standard (1, 6, 7, 10, 13, 15, 23, 26–29), 20 for CHA (1, 3–5, 8–10, 13, 15–22, 24–26, 29), and 3 for the “CHA & Lepidic” criteria (5, 18, 26).

### Data Characteristics and Quality Assessment

**Figure 2:**
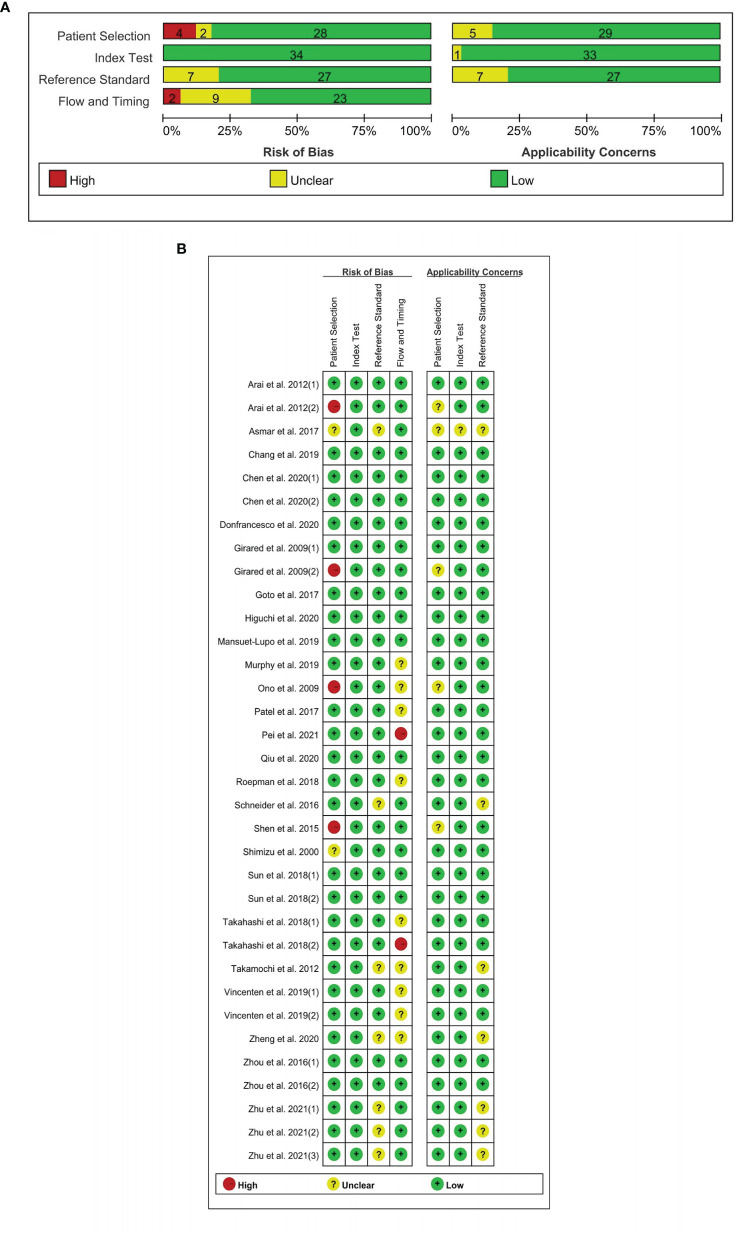
Quality of the selected studies according to the QUADAS-2 guidelines. **(A)** Risk of bias graph. **(B)** Risk of summary.

**Table 1** shows the detailed features of the 34 included articles that were classified by the first author’s initials (ranging from A to Z). The meta-analysis takes tumor pairs as a unit, owing to the fact that identifying the relationship among various tumors is of great clinical significance. A total of 1,075 pairs of tumors were included in the 34 studies. Twenty-five studies mentioned double and multiple primary lung cancer (i.e., cancer type was multiple), while nine involved only double primary lung cancer (i.e., cancer type was dual). As shown in **Figure 2**, the QUADAS-2 checklist indicated that the quality of the selected studies was moderate to high.

**Table 1:** Summary of the 34 studies included in the meta-analysis.

First author (year)	Country	Cancer type	Tumor pairs (MPLC/IPM)	Method	TP	FP	FN	TN	Sen (%)	Spe (%)	Con (%)
Arai (2012) (1) (1)	Japan	Dual	12 (6/6)	CHA	5	1	1	5	83.3%	83.3%	83.3%
Arai (2012) (2) (1)	Japan	Dual	12 (6/6)	M-M	5	3	1	3	83.3%	50.0%	66.7%
Asmar (2017) (16)	USA	Multiple	87 (67/20)	CHA	51	7	16	13	76.1%	65.0%	73.6%
Chang (2019) (17)	USA	Multiple	76 (51/25)	CHA	45	11	6	14	88.2%	56.0%	77.6%
Chen (2020) (1) (18)	China	Multiple	19 (14/5)	CHA	12	2	2	3	85.7%	60.0%	78.9%
Chen (2020) (2) (18)	China	Multiple	19 (14/5)	CHA & Lepidic	14	3	0	2	100.0%	40.0%	84.2%
Donfrancesco (2020) (19)	France	Multiple	24 (17/7)	CHA	12	1	5	6	70.6%	85.7%	75.0%
Girard (2009) (13)	USA	Multiple	22 (14/8)	CHA	13	1	1	7	92.9%	87.5%	90.9%
Girard (2009) (13)	USA	Multiple	22 (14/8)	M-M	13	6	1	2	92.9%	25.0%	68.2%
Goto (2017) (20)	Japan	Dual	12 (11/1)	CHA	9	1	2	0	81.8%	0.0%	75.0%
Higuchi (2020) (21)	Japan	Multiple	39 (31/8)	CHA	29	4	2	4	93.5%	50.0%	84.6%
Mansuet-Lupo (2019) (3)	France	Dual	109 (70/39)	CHA	50	10	20	29	71.4%	74.4%	72.5%
Murphy (2019) (22)	USA	Multiple	34 (26/8)	CHA	24	0	2	8	92.3%	100.0%	94.1%
Ono (2009) (7)	Japan	Multiple	70 (45/25)	M-M	41	9	4	16	91.1%	64.0%	81.4%
Patel (2017) (8)	USA	Multiple	16 (13/3)	CHA	13	2	0	1	100.0%	33.3%	87.5%
Pei (2021) (23)	China	Multiple	30 (26/4)	M-M	15	3	11	1	57.7%	25.0%	53.3%
Qiu (2019) (24)	China	Dual	34 (9/25)	CHA	9	3	0	22	100.0%	88.0%	91.2%
Roepman (2018) (4)	Netherlands	Multiple	43 (34/9)	CHA	23	0	11	9	67.6%	100.0%	74.4%
Schneider (2016) (9)	USA	Multiple	27 (15/12)	CHA	7	5	8	7	46.7%	58.3%	51.9%
Shen (2015) (27)	China	Dual	12 (5/7)	M-M	4	1	1	6	80.0%	85.7%	83.3%
Shimizu (2000) (28)	Japan	Dual	14 (1/13)	M-M	1	2	0	11	100.0%	84.6%	85.7%
Sun (2018) (1) (5)	China	Multiple	20 (12/8)	CHA	8	3	4	5	66.7%	62.5%	65.0%
Sun (2018) (2) (5)	China	Multiple	20 (12/8)	CHA & Lepidic	12	3	0	5	100.0%	62.5%	85.0%
Takamochi (2012) (6)	Japan	Multiple	50 (36/14)	M-M	31	14	5	0	86.1%	0.0%	62.0%
Takahashi (2018) (1) (10)	Japan	Multiple	20 (13/7)	CHA	5	1	8	6	38.5%	85.7%	55.0%
Takahashi (2018) (2) (10)	Japan	Multiple	32 (12/20)	M-M	11	19	1	1	91.7%	5.0%	37.5%
Vincenten (2019) (1) (15)	Netherlands	Multiple	34 (10/24)	CHA	4	7	6	17	40.0%	70.8%	61.8%
Vincenten (2019) (2) (15)	Netherlands	Multiple	34 (10/24)	M-M	7	14	3	10	70.0%	41.7%	50.0%
Zheng (2020) (25)	China	Multiple	18 (14/4)	CHA	8	1	6	3	57.1%	75.0%	61.1%
Zhou (2016) (1) (29)	China	Dual	24 (8/16)	CHA	3	0	5	16	37.5%	100.0%	79.2%
Zhou (2016) (2) (29)	China	Dual	24 (8/16)	M-M	4	3	4	13	50.0%	81.3%	70.8%
Zhu (2021) (1) (26)	China	Multiple	22 (20/2)	CHA	16	2	4	0	80.0%	0.0%	72.7%
Zhu (2021) (2) (26)	China	Multiple	22 (20/2)	M-M	11	0	9	2	55.0%	100.0%	59.1%
Zhu (2021) (3) (26)	China	Multiple	22 (20/2)	CHA & Lepidic	18	2	2	0	90.0%	0.0%	81.8%

Dual: only two pairs of tumors. Multiple: two or more pairs of tumors.

Sen, sensitivity; Spe, specificity; Con, consistency.

### Heterogeneity Analysis

**Figure 3:**
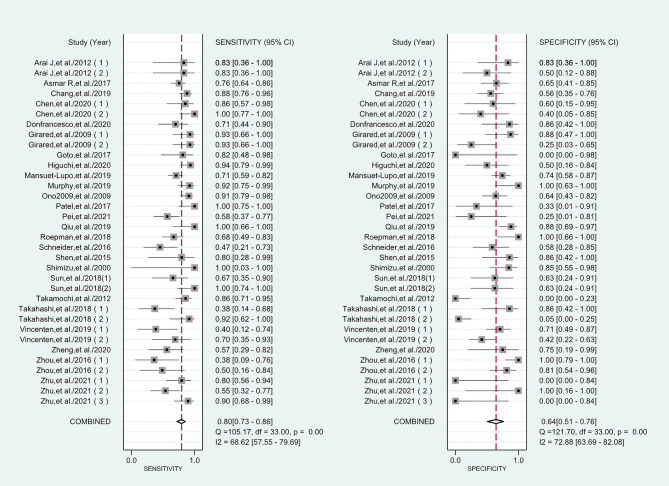
Forest plots of sensitivities and specificities for histology in the differential diagnosis of multiple primary lung cancer (MPLC) and intrapulmonary metastasis (IPM).

**Figure 4:**
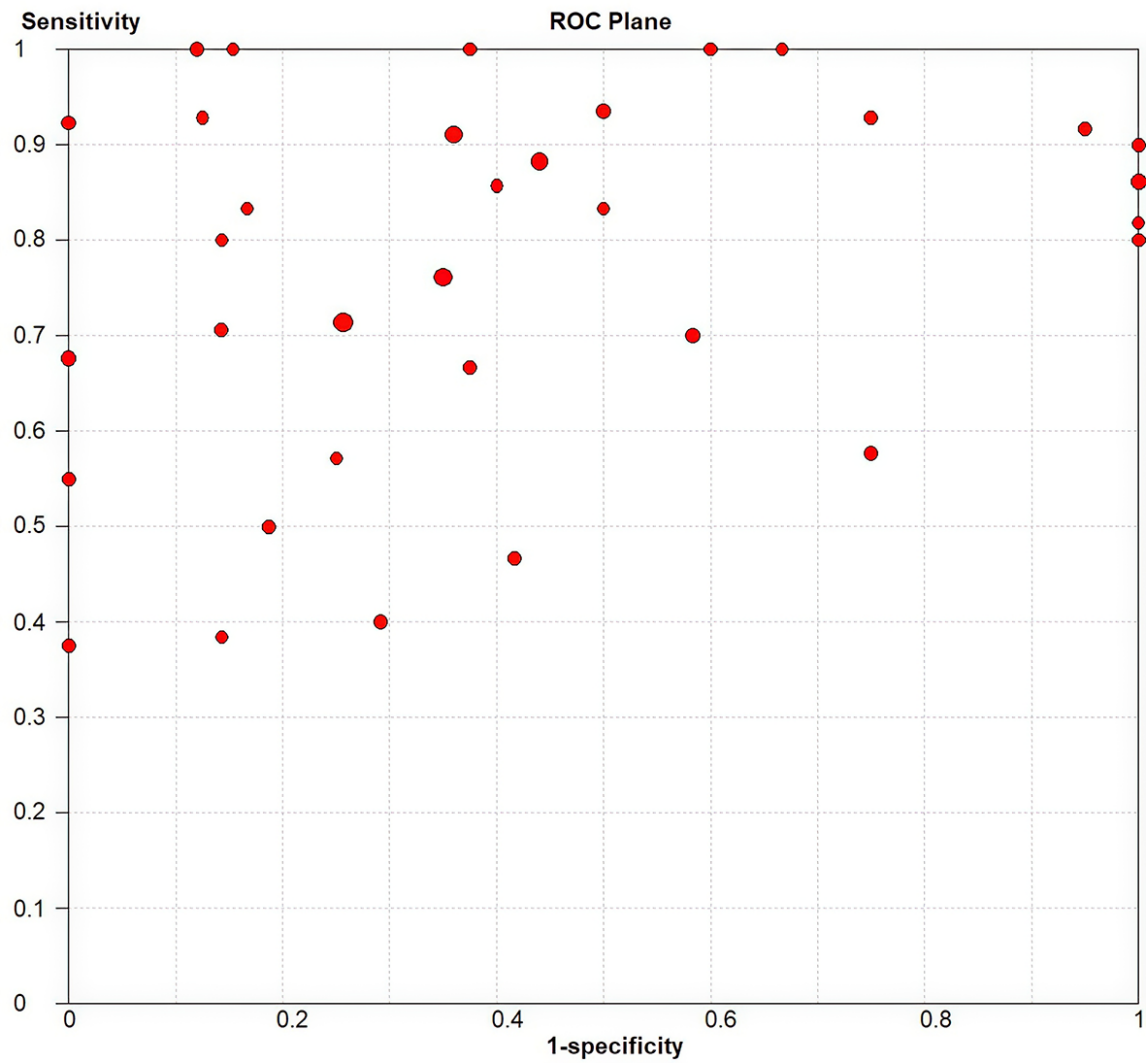
The ROC plane for assessing threshold effects.

**Figure 5:**
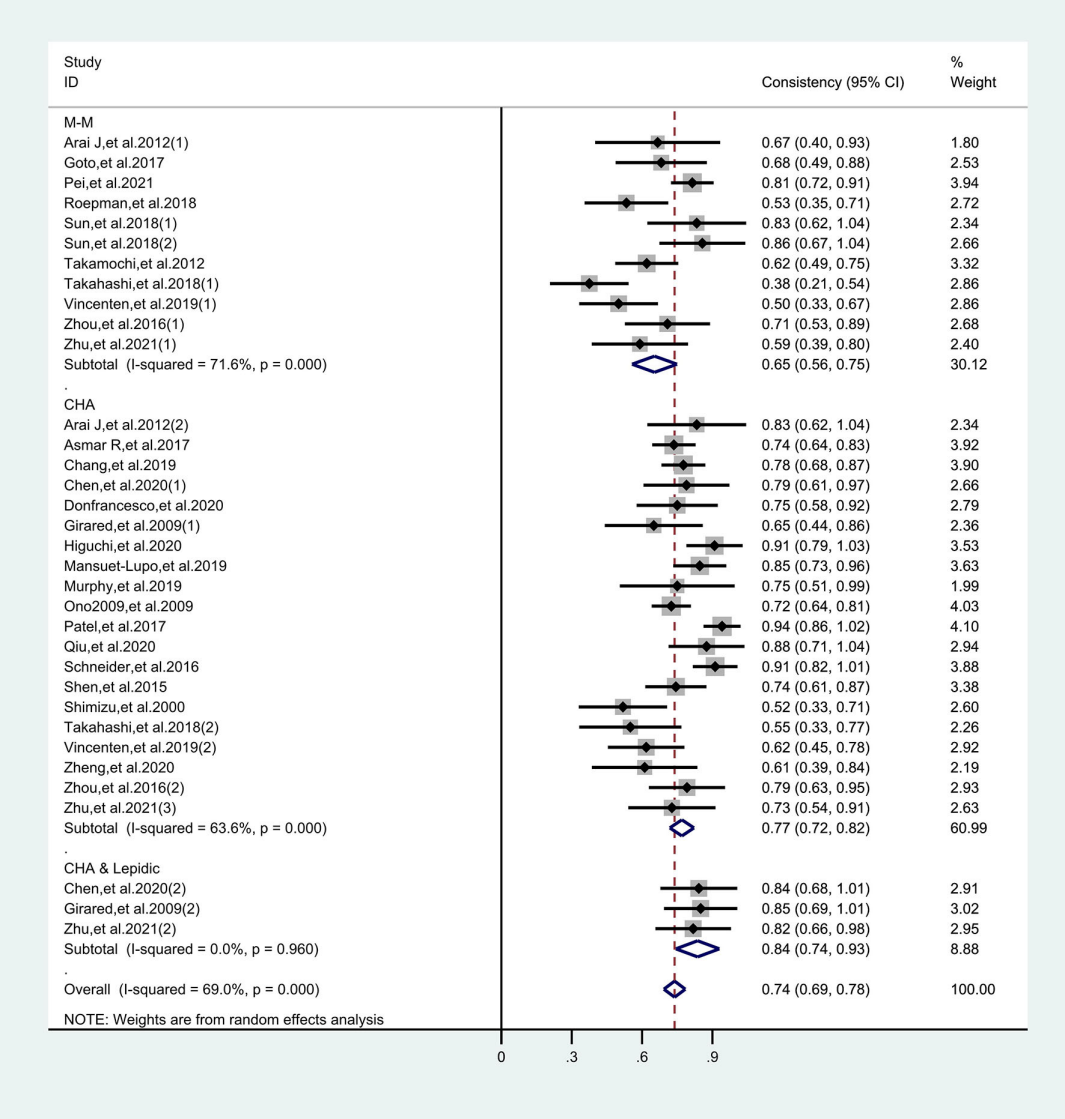
Forest plots of consistency for each histological method in the differential diagnosis of MPLC and IPM.

*I* ^2^ values of the pooled sensitivity and specificity for all of the studies were 68.62% (95% CI: 57.55–79.69) and 72.88% (95% CI: 63.69–82.08), respectively (**Figure 3**), indicating medium levels of heterogeneity in sensitivity and specificity. Hence, we explored the origin of potential heterogeneity. However, the ROC plane generated by Meta-DiSc 1.4 software did not present a “shoulder-arm” shape (**Figure 4**). In addition, the *P*-value of the Spearman correlation coefficient was found to be 0.317 (*P* = 0.068). The aforesaid results provided evidence that heterogeneity did not originate from the threshold effect. Meta-regression analysis and subgroup analysis were adopted to confirm if the heterogeneity was caused by the non-threshold effect. Cancer type, histological method, quantity, and continent were used as covariants in meta-regression based on the different characteristics of each study. As shown in **Table 2**, the data indicated that all covariants did not explain the heterogeneity (*P* > 0.05). Therefore, the random-effects model was adopted in this meta-analysis to eliminate the impact of heterogeneity on the results (32). Subgroup analysis based on the histological method revealed that the pooled sensitivity, specificity, and consistency rate in the 11 studies related to the M-M standard were 0.78 (95% CI: 0.71–0.84), 0.47 (95% CI: 0.38–0.55), and 65% (95% CI: 0.56–0.75), respectively; for the 20 studies associated with CHA, these values were 0.76 (95% CI: 0.72–0.80), 0.74 (95% CI: 0.68–0.79), and 77% (95% CI: 0.72–0.82), respectively; and for the 3 studies that involved the “CHA & Lepidic” criteria, these values were 0.96 (95% CI: 0.85–0.99), 0.47 (95% CI: 0.21–0.73), and 84% (95% CI: 0.74–0.93), respectively (**Table 3** and **Figure 5**).

**Table 2:** RDOR and *P*-values of covariants in the meta-regression analysis.

Var	RDOR	95% CI	*P*-value
Type	0.99	(0.29, 3.41)	0.99
Method	1.89	(0.71, 5.03)	0.19
Quantity	0.74	(0.22, 2.51)	0.74
Continent	1.09	(0.31, 3.80)	1.09

**Table 3:** Summary results of the subgroup analysis for histology in the differential diagnosis of MPLC and IPM.

Subtype	Number of studies	Sensitivity (95% CI)	Specificity (95% CI)	PLR (95% CI)	NLR (95% CI)	DOR (95% CI)
Method						
M-M	11	0.78 (0.71–0.84)	0.47 (0.38–0.55)	1.42 (0.98–2.06)	0.46 (0.32–0.68)	3.37 (2.00–5.69)
CHA	20	0.76 (0.72–0.80)	0.74 (0.68–0.79)	2.53 (2.04–3.13)	0.40 (0.30–0.54)	7.33 (5.12–10.48)
CHA & Lepidic	3	0.96 (0.85–0.99)	0.47 (0.21–0.73)	1.71 (1.13–2.59)	0.12 (0.03–0.56)	12.37 (2.78–55.08)
Continent						
Asia	22	0.80 (0.70–0.87)	0.61 (0.40–0.78)	2.04 (1.26–3.29)	0.33 (0.21–0.50)	6.23 (2.78–13.97)
Europe or America	12	0.79 (0.68–0.87)	0.68 (0.54–0.80)	2.48 (1.63–3.77)	0.31 (0.19–0.49)	8.05 (3.71–17.44)
Quantity						
<30	20	0.79 (0.69–0.87)	0.70 (0.56–0.81)	2.68 (1.83–3.92)	0.29 (0.20–0.43)	9.16 (5.12–16.41)
≥30	14	0.80 (0.70–0.87)	0.58 (0.35–0.78)	1.92 (1.10–3.37)	0.34 (0.19–0.60)	5.63 (1.95–16.29)
Type						
Dual	9	0.71 (0.56–0.82)	0.79 (0.67–0.88)	3.40 (2.02–5.73)	0.37 (0.23–0.59)	9.16 (3.81–22.03)
Multiple	25	0.82 (0.75–0.88)	0.58 (0.41–0.73)	1.95 (1.34–2.84)	0.31 (0.20–0.46)	6.40 (3.19–12.83)

### Differential Diagnostic Value of Histology

**Figure 6:**
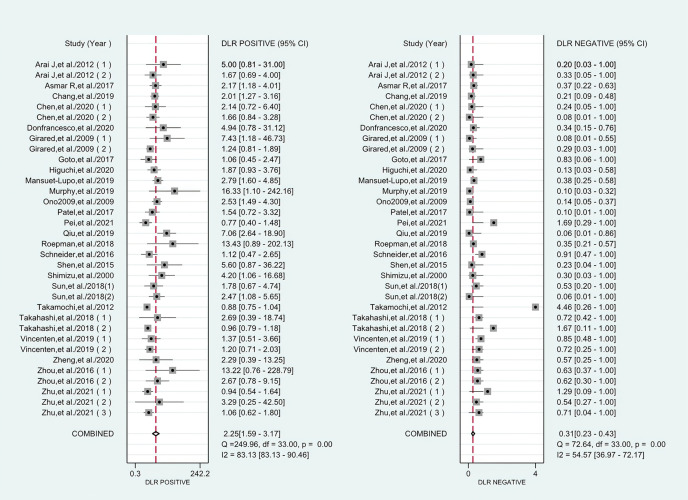
Forest plots of PLR and NLR for histology in the differential diagnosis of MPLC and IPM.

**Figure 7:**
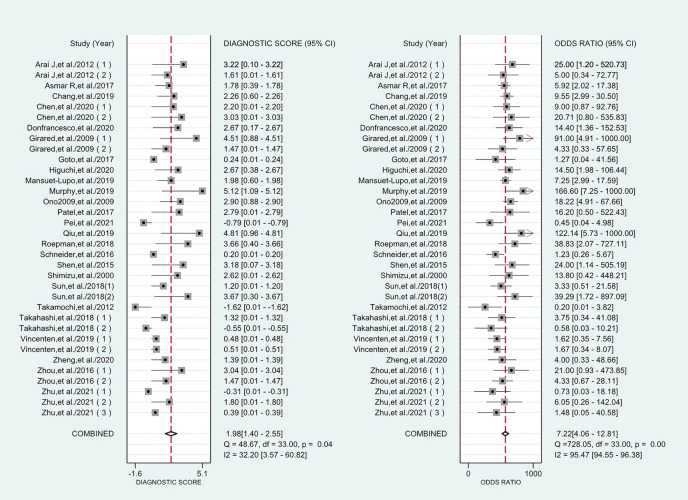
Forest plots of the diagnostic score and DOR for histology in the differential diagnosis of MPLC and IPM.

**Figure 8:**
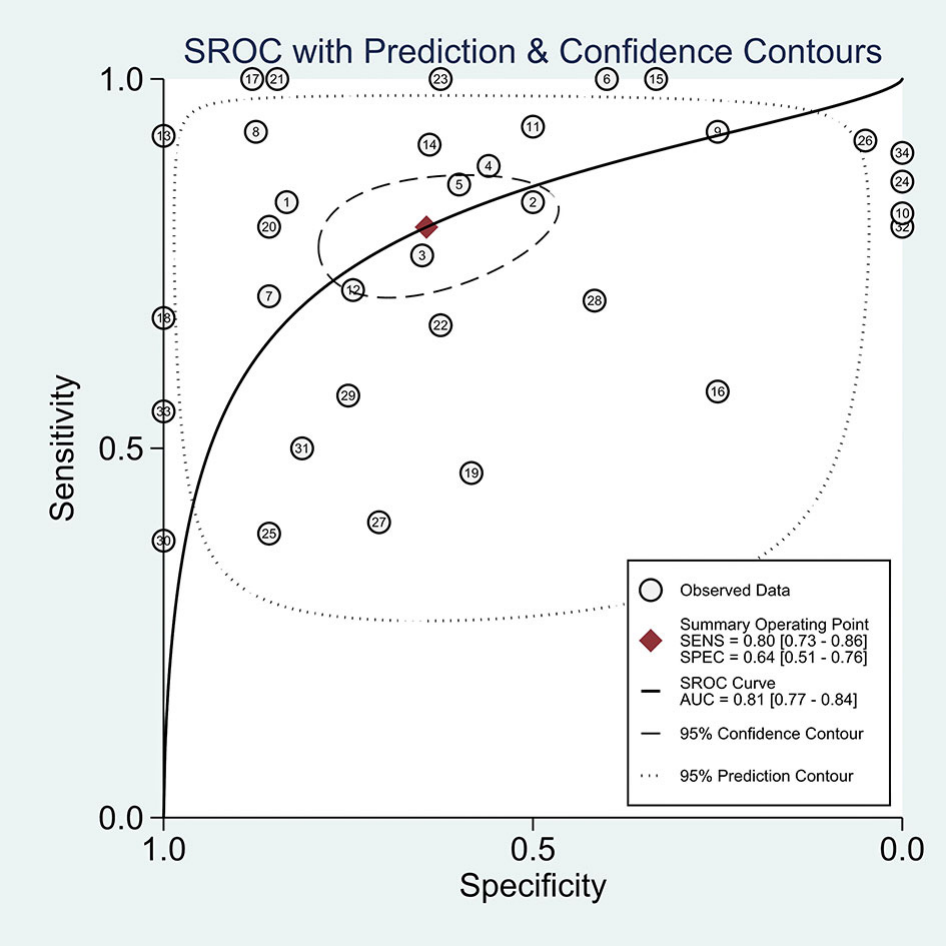
The SROC curve of the differential diagnostic value of histology in MPLC and IPM.

**Figure 9:**
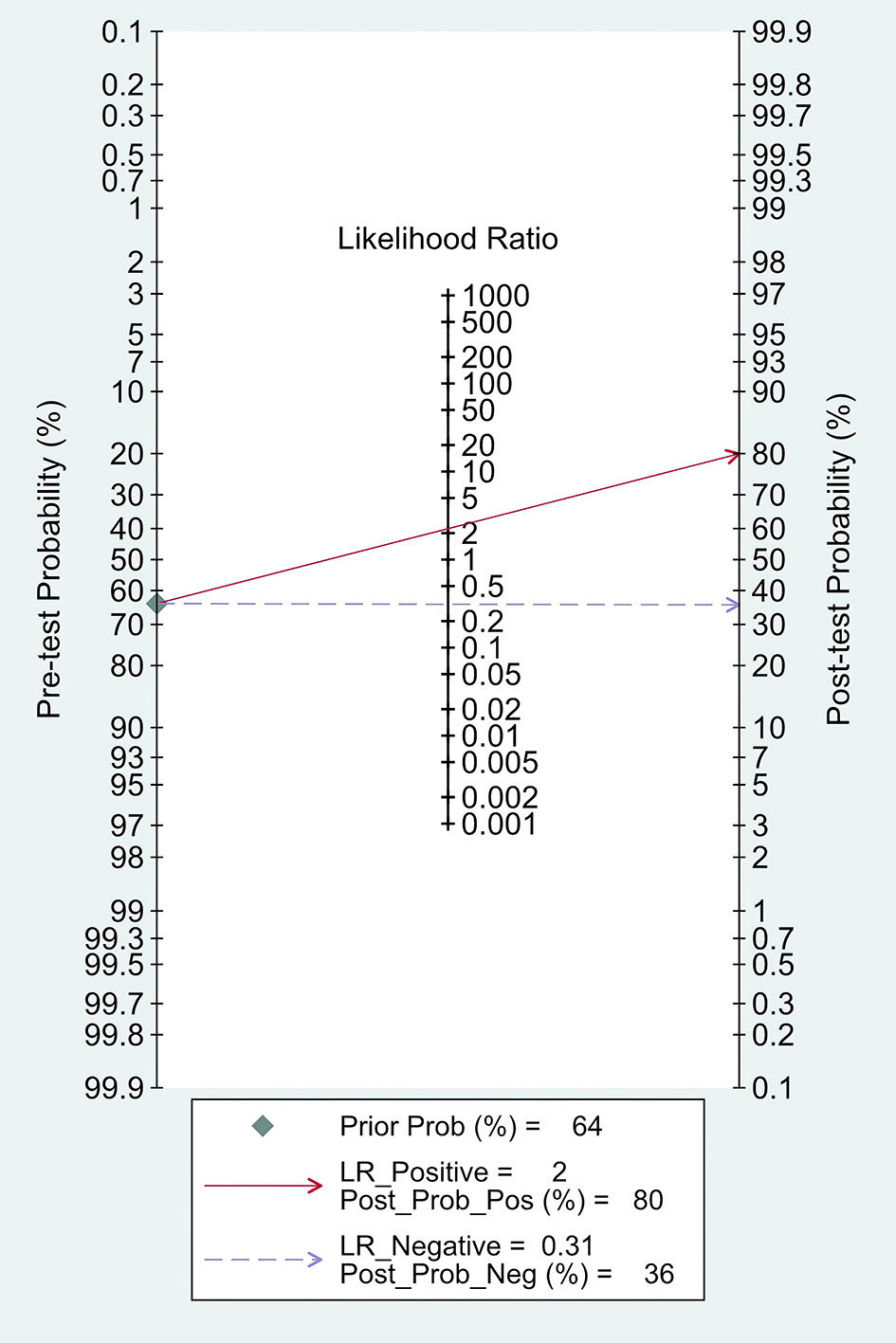
Fagan’s nomogram for likelihood ratios.

**Figure 10:**
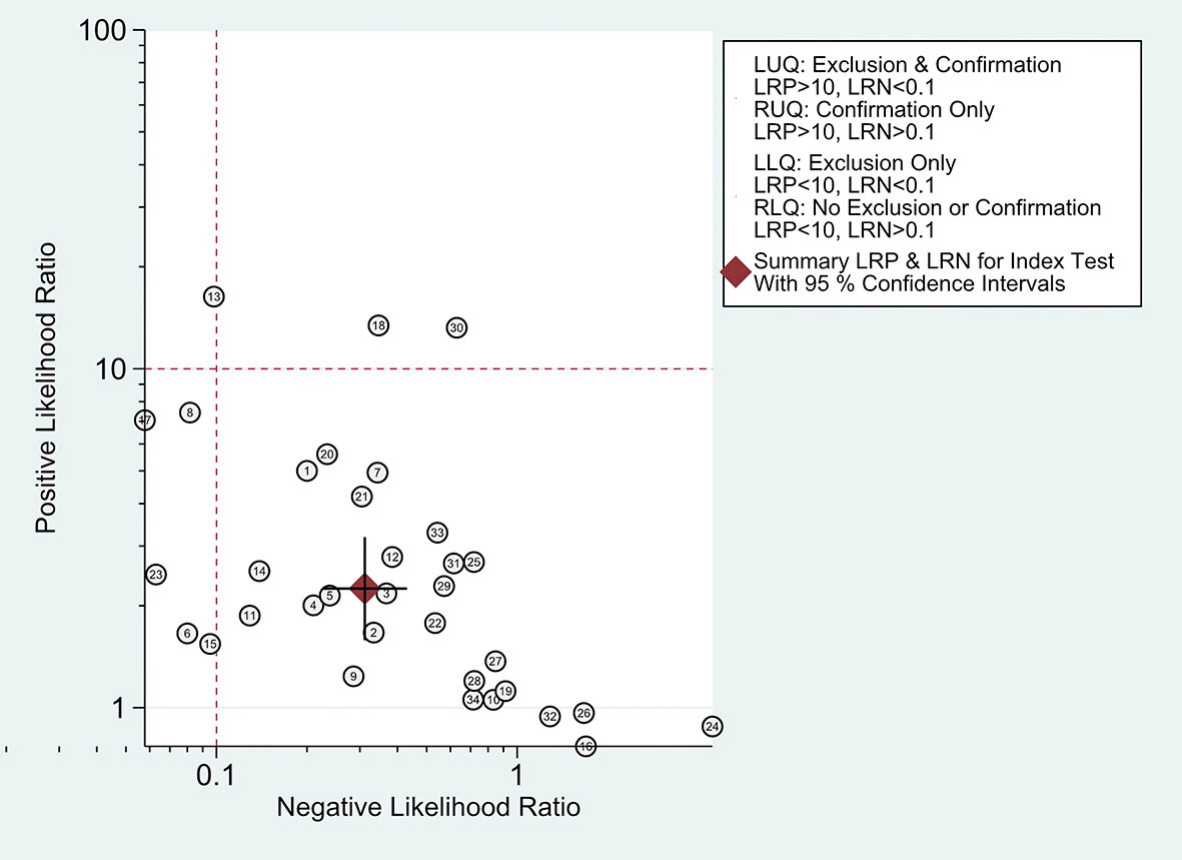
The likelihood ratio scattergram.

**Figure 11:**
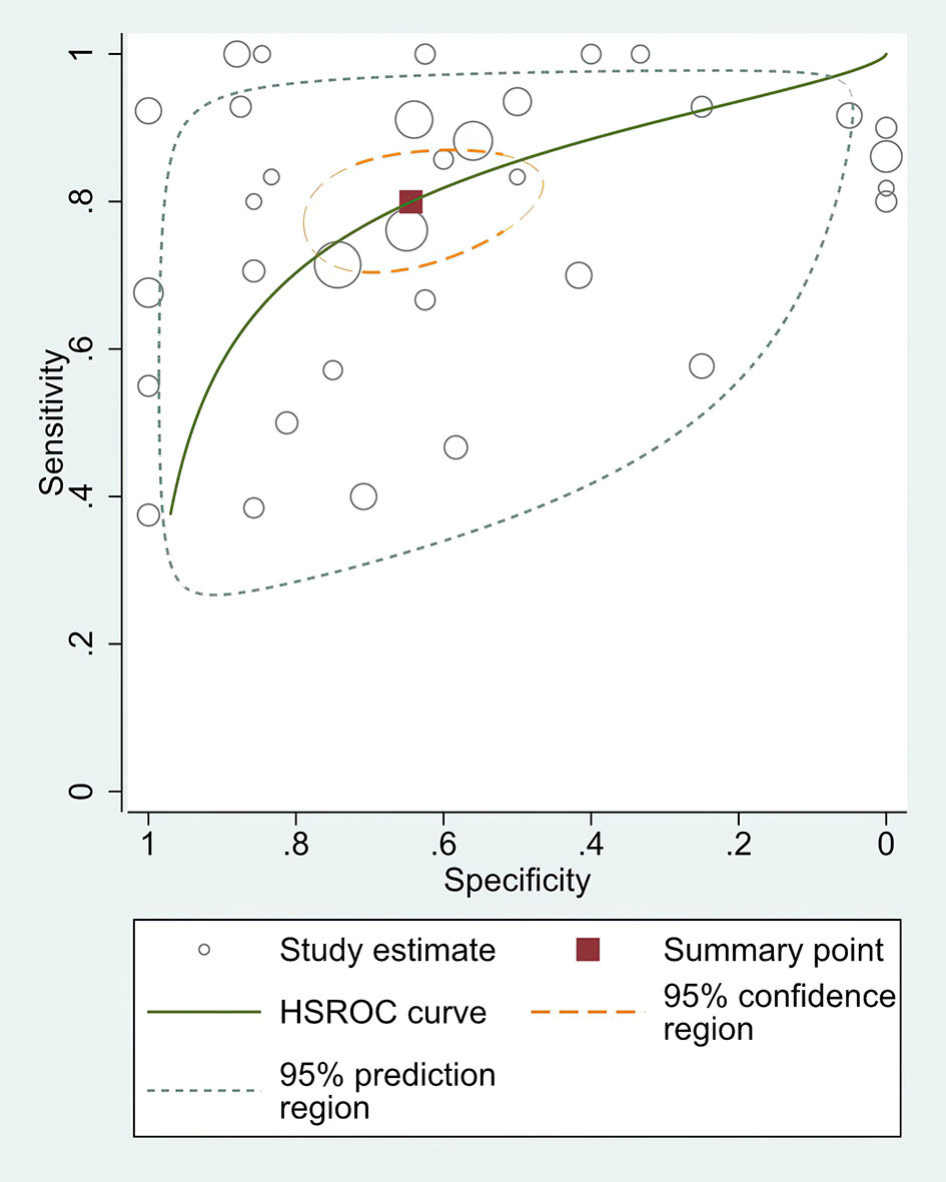
The HSROC curve of the differential diagnostic value of histology in MPLC and IPM.

The combined pooled sensitivity, specificity, positive likelihood ratio (PLR), negative likelihood ratio (NLR), diagnostic odds ratio (DOR), consistency rate, and the area under the summary receiver operator characteristic (SROC) curve of the 34 studies were 0.80 (95% CI: 0.73–0.86), 0.64 (95% CI: 0.51–0.76), 2.25 (95% CI: 1.59–3.17), 0.31 (95% CI: 0.23–0.43), 7.22 (95% CI: 4.06–12.81), 74% (95% CI: 0.69–0.78), and 0.81 (95% CI: 0.77–0.84), respectively (**Figures 3**, **5**–**8**). **Figure 9** shows Fagan’s nomogram for the assessment of posttest probabilities resulting from different pretest probabilities. Given a pretest probability of 64% on the basis of the prevalence rates of our own practice population, the posttest probability rates of MPLC and IPM were 80% and 36%, respectively. **Figure 10** presents a scattergram for PLR and NLR, which was utilized to determine the clinical values of different diagnostic methods and defined quadrants of informativeness based on established evidence-based thresholds: the left upper quadrant [likelihood ratio positive (LRP) > 10, likelihood ratio negative (LRN) < 0.1] presents both exclusion and confirmation, the right upper quadrant (LRP > 10, LRN > 0.1) confirmation only, the left lower quadrant (LRP < 10, LRN < 0.1) exclusion only, and the right lower quadrant (LRP < 10, LRN > 0.1) neither confirmation nor exclusion (33). One of the 34 studies was located in the left upper quadrant, two were in the right upper quadrant, five were in the left lower quadrant, and the remaining studies were in the right lower quadrant. An HSROC curve was performed in **Figure 11**. The estimated value of *β* was 0.42 (95% CI: −0.13 to 0.98), and the value of *z* and the *P*-value were 1.49 and 0.14 separately, signifying that the SROC curve was symmetric. In addition, the value of Lambda was 2.19 (95% CI: 1.57–2.80). The aforesaid results suggested that histology had a moderate differential diagnostic value between MPLC and IPM.

### Sensitivity Analysis

We conducted sensitivity analyses to evaluate the influence of each study on the outcome of the meta-analysis (**Table 4**). Nevertheless, the pooled DOR showed less variation, which indicated that the stability of the included literature was acceptable.

**Table 4:** The influence of each study on the outcome of the meta-analysis.

First author (year)	DOR	95% CI
Arai (2012) (1) (1)	6.98	3.89–12.52
Arai (2012) (2) (1)	7.32	4.04–13.25
Asmar (2017) (16)	7.35	4.00–13.53
Chang (2019) (17)	7.12	3.88–13.06
Chen (2020) (1) (18)	7.17	3.96–13.00
Chen (2020) (2) (18)	7.03	3.91–12.63
Donfrancesco (2020) (19)	7.09	3.92–12.84
Girard (2009) (13)	6.68	3.78–11.79
Girard (2009) (13)	7.40	4.11–13.32
Goto (2017) (20)	7.46	4.18–13.34
Higuchi (2020) (21)	7.00	3.86–12.69
Mansuet-Lupo (2019) (3)	7.30	3.96–13.44
Murphy (2019) (22)	6.40	3.75–10.94
Ono (2009) (7)	6.88	3.78–12.50
Patel (2017) (8)	7.08	3.95–12.69
Pei (2021) (23)	7.86	4.47–13.81
Qiu (2019) (24)	6.51	3.75–11.31
Roepman (2018) (4)	6.86	3.86–12.19
Schneider (2016) (9)	7.69	4.29–13.80
Shen (2015) (27)	6.97	3.89–12.50
Shimizu (2000) (28)	6.93	3.87–12.39
Sun (2018) (1) (5)	7.43	4.08–13.52
Sun (2018) (2) (5)	6.84	3.82–12.27
Takamochi (2012) (6)	8.06	4.74–13.68
Takahashi (2018) (1) (10)	7.32	4.05–13.24
Takahashi (2018) (2) (10)	7.83	4.54–13.52
Vincenten (2019) (1) (15)	7.56	4.17–13.70
Vincenten (2019) (2) (15)	7.66	4.24–13.83
Zheng (2020) (25)	7.36	4.07–13.33
Zhou (2016) (1) (29)	6.91	3.89–12.28
Zhou (2016) (2) (29)	7.29	4.00–13.28
Zhu (2021) (1) (26)	7.66	4.30–13.63
Zhu (2021) (2) (26)	7.21	4.01–12.96
Zhu (2021) (3) (26)	7.47	4.18–13.33
Combined	7.22	4.06–12.81

### Publication Bias

**Figure 12:**
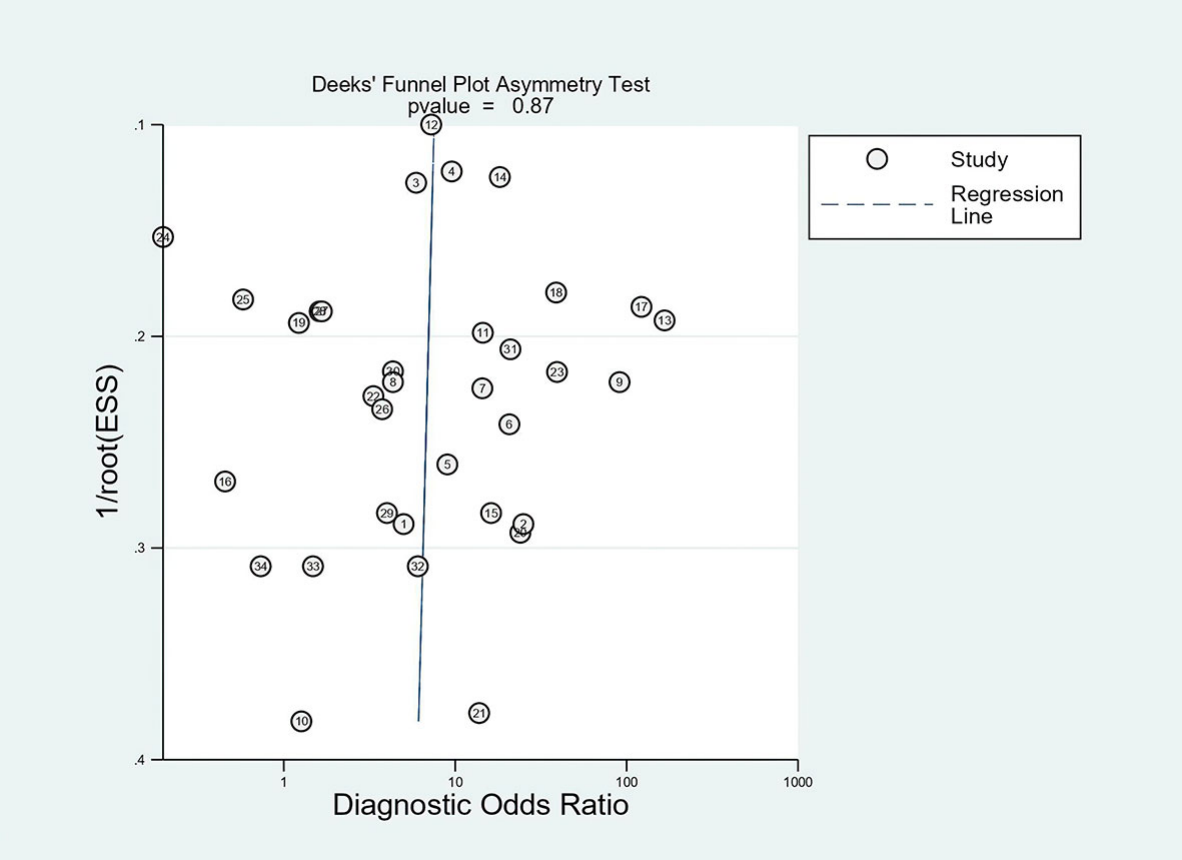
The result of Deeks’ funnel test.

The publication bias of the studies was assessed by using Deeks’ funnel plot asymmetry (**Figure 12**). The *P*-value for the linear regression was 0.87, implying that there was no significant publication bias in this meta-analysis.

## Discussion

The position of histology in the differential diagnosis between MPLC and IPM has been greatly challenged since the advent of molecular analysis. However, it is still the major method in the discrimination of MPLC and IPM in the clinical setting due to its convenience, economy, and utility. Furthermore, a comprehensive and systematic assessment regarding histological clinical value is lacking. As a result, after collecting a sufficient sample size required for the study, a meta-analysis was performed to estimate the differential diagnostic value of the M-M standard, CHA, “CHA & Lepidic” criteria, and overall histology in MPLC and IPM.

This meta-analysis included 34 studies performed between 2000 and 2021 involving a total of 1,075 pairs of tumors. Here, the area under the SROC curve was found to be 0.81, implying that histology had a moderate differential diagnostic value between MPLC and IPM (34). The pooled sensitivity and specificity of the 34 studies combined were 0.80 (95% CI: 0.73–0.86) and 0.64 (95% CI: 0.51–0.76), respectively. In addition, we found that the pooled DOR was 7.22, suggesting that histology was definitely a useful differential diagnostic method for MLC patients (35). The aforesaid findings were also further verified *via* the HSROC model. When considered together, these data indicate a moderate value of histology in the distinction of MPLC from IPM.

Heterogeneity, a factor that must be considered, was used to explain the results of the meta-analysis. In this study, there was medium heterogeneity in sensitivity and specificity. However, meta-regression did not show the source of this heterogeneity. Afterward, subgroup analyses by cancer type, histological method, quantity, and continent were implemented to confirm the factors that accounted for this heterogeneity, but they failed. It is noteworthy that the histological method contributed the most to heterogeneity in the meta-regression analysis. In detail, the histological method yielded maximal RDOR value (i.e., 1.89) and minimal *P*-value (i.e., 0.19) among all covariates. In addition, in the subgroup analysis, the pooled sensitivity, specificity, and consistency rate in the 11 studies based on the M-M standard were 0.78, 0.47, and 65%, respectively; for the 20 studies related to CHA, these values were 0.76, 0.74, and 77%, respectively; and for the 3 studies that consisted of the “CHA & Lepidic” criteria, these values were 0.96, 0.47, and 84%, respectively. The M-M standard had similar sensitivity but poor specificity compared with CHA. The variation may be interpreted as follows: a) the M-M standard was proposed on the basis of tumor locations, histological characteristics, and lymph node metastasis. Mixed histological features are manifested in more than 80% of patients with lung adenocarcinoma (36) and are arduous to differentiate using the M-M standard. Unlike IPM, finding similarities is not sufficient for diagnosing MPLC (37). CHA presents a promising procedure for resolving the aforesaid dilemma to some degree as it considers that an individual tumor is provided with distinctive histologic characteristics such as cytologic features, stromal characteristics, and associated inflammatory milieu; b) IPM is defined as tumors that have similar histology with the primary tumor based on the M-M criteria. However, multiple squamous cell cancers in the fibrotic lung sometimes arise within the same area. Moreover, bronchioloalveolar carcinomas commonly manifest multiple ground-glass attenuations within the same segments, and thus, these are usually defined as MPLC (9). c) MLC with nodal invasion is classified as IPM according to the M-M criteria. However, lymph node status is not invariably conducive to classifying MLC. Mansuet-Lupo et al. (3) found that 20 patients with MPLC had node involvement and 13 patients with IPM were N0 (i.e., no lymph node metastasis). In the subgroup analysis of the performance of the three histological methods, the “CHA & Lepidic” criteria yielded the highest overall sensitivity and consistency rate, which might be attributed to the idea that this novel standard took into account the diagnostic value of lepidic. Specifically, apart from CHA, tumors with low-grade lepidic component were also defined as MPLC (5). Although lepidic growth can arise in IPM, they are usually mucinous (38) with severe atypia (39). In addition, a non-mucinous lepidic component with mild atypia is a favorable prognostic factor in MLC (14). It suggests that tumors with a low-grade lepidic component prefer MPLC. However, some studies indicated that lepidic architecture was not reproducible in the multiobserver study and, thus, might not be accurate enough to differentiate MPLC from IPM (19, 40). Unfortunately, correlative reports were few; thus, there were only three articles to perform this meta-analysis. These results should be interpreted with caution due to the few included studies. More standardized research on the “CHA & Lepidic” standard is needed in the future.

Several important limitations of histology in the differential diagnosis of MPLC and IPM should be considered as well. First, although in a substantial number of cases, histologic patterns can be preserved, the problem of histologic progression in a handful of cases exhibits a limitation to histology-based definition of tumor relationship (41), and thus, IPM is incorrectly predicted to be MPLC. Additionally, histologic assessment is subjective with interobserver variability and may lead to a different conclusion. It was reported that the reproducibility of histological subtyping between different pathologists was only fair to moderate (40). The study conducted by Murphy et al. showed that although histologic evaluation was performed independently by two experienced pathologists, 7 (17.1%) of 41 pairs of tumors were still indeterminate (22). Hence, a comprehensive assessment combined with the actual circumstance of patients should be carried out in the clinical field.

This meta-analysis had some significant limitations requiring attention when interpreting the results. First, some data, such as non-English studies, conference abstracts, editorials, guidelines, and other unpublished literature online, were excluded for the improvement of literature quality. All may inevitably increase publication bias to a certain extent, although there was no significant publication bias according to the results of Deeks’ funnel test. Second, although 34 studies were included, the overall sample size was still small, so the significance of the present results was limited. Third, medium heterogeneity was observed in the pooled sensitivity and specificity due to the diversity of sensitivity and specificity reported among all of the studies. Nevertheless, we had already realized this before we performed this meta-analysis and used subgroup and meta-regression analyses to explore the origin of potential heterogeneity and sensitivity analysis to confirm the stability of the pooled estimates.

## Conclusion

In conclusion, we found that histology had a moderate differential diagnostic value, which was still the major method of differential diagnosis between MPLC and IPM, thanks to its availability, cost, and turnaround times. In addition, molecular diagnosis was recommended if conditions allowed. In these three subtypes of histology, CHA had a better differential diagnostic value compared with the M-M standard. In addition, the “CHA & Lepidic” criteria yielded the highest sensitivity and showed great application potential. However, further studies are needed to verify these findings.

## Data Availability Statement

The original contributions presented in the study are included in the article/**Supplementary Material**. Further inquiries can be directed to the corresponding authors.

## Author Contributions

Concept and design: ST, FL, JP, RC, and CB. Literature search: FL, JP, and HS. Data extraction and quality assessment: ST, FL, and YZ. Statistical analysis: ST and YZ. Manuscript writing: ST, FL, and JP. Administrative support: YD, RC, and CB. Final approval of the manuscript: all authors.

## Funding

This work was supported by the Collaborative Innovation Cluster Project of Shanghai Municipal Health Commission (No. 2020CXJQ03) and the Clinical Research Plan of SHDC (No. SHDC2020CR1021B-005). The funders of the study had no role in the concept, design, literature search, data extraction, quality assessment, statistical analysis, manuscript writing, administrative support, or writing of the manuscript.

## Conflict of Interest

The authors declare that the research was conducted in the absence of any commercial or financial relationships that could be construed as a potential conflict of interest.

## Publisher’s Note

All claims expressed in this article are solely those of the authors and do not necessarily represent those of their affiliated organizations, or those of the publisher, the editors and the reviewers. Any product that may be evaluated in this article, or claim that may be made by its manufacturer, is not guaranteed or endorsed by the publisher.

## Abbreviations

MPLC, multiple primary lung cancer; IPM, intrapulmonary metastasis; MLC, multiple lung cancer; PRISMA, Preferred Reporting Items for Systematic Reviews and Meta-Analyses; M-M, Martini and Melamed; CHA, comprehensive histologic assessment; PLR, positive likelihood ratio; NLR, negative likelihood ratio; DOR, diagnostic odds ratio; SROC, summary receiver operator characteristic; HRCT, high-resolution computed tomography; aCGH, array-based comparative genomic hybridization; NGS, next-generation sequencing; MSI, microsatellite instability; AUC, area under the curve; AAH, atypical adenomatous hyperplasia; AIS, adenocarcinoma *in situ*; MeSH, Medical Subject Headings; ROC, receiver operator characteristic; TP, true positive; FP, false positive; TN, true negative; FN, false negative; RDOR, relative diagnostic odds ratio; CI, confidence interval; LRP, likelihood ratio positive; LRN, likelihood ratio negative; HSROC, hierarchical summary receiver operating characteristic.
