# Supplementary material for: Differential Diagnostic Value of Histology in MPLC and IPM: A Systematic Review and Meta-Analysis
Source: Front Oncol. 2022 Apr 29;12:871827. doi: 10.3389/fonc.2022.871827 (PMC9099226; doi:10.3389/fonc.2022.871827)
Supplement: Supplementary file 1 [file Table_1.docx]

**Supplementary Table: The detailed search strategy**

**PubMed**

#1 synchronous [Title/Abstract]

#2 multiple [Title/Abstract]

#3 separate [Title/Abstract]

#4 multifocal [Title/Abstract]

#5 OR/ (#1-4)

#6 "lung cancer"[Title/Abstract]

#7 "lung cancers"[Title/Abstract]

#8 "lung carcinoma"[Title/Abstract]

#9 "lung carcinomas"[Title/Abstract]

#10 "Lung Neoplasms"[MeSH Terms]

#11 OR/ (#6-10)

#12 molecular [Title/Abstract]

#13 genomic [Title/Abstract]

#14 "next-generation sequencing"[Title/Abstract]

#15 "protein expression"[Title/Abstract]

#16 OR/ (#12-15)

#17 #5 AND #11 AND #16

**EMBASE**

#1 TI= (synchronous) OR AB= (synchronous)

#2 TI= (multiple) OR AB= (multiple)

#3 TI= (separate) OR AB= (separate)

#4 TI= (multifocal) OR AB= (multifocal)

#5 OR/ (#1-4)

#6 TI= ("lung cancer") OR AB= ("lung cancer")

#7 TI= ("lung cancers") OR AB= ("lung cancers")

#8 TI= ("lung carcinoma") OR AB= ("lung carcinoma")

#9 TI= ("lung carcinomas") OR AB= ("lung carcinomas")

#10 TS= ("Lung Neoplasms")

#11 OR/ (#6-10)

#12 TI= (molecular) OR AB= (molecular)

#13 TI= (genomic) OR AB= (genomic)

#14 TI= ("next-generation sequencing") OR AB= ("next-generation sequencing")

#15 TI= ("protein expression") OR AB= ("protein expression")

#16 OR/ (#12-15)

#17 #5 AND #11 AND #16

**Web of Science**

#1 synchronous: ab,ti

#2 multiple: ab,ti

#3 separate: ab,ti

#4 multifocal: ab,ti

#5 OR/ (#1-4)

#6 'lung tumor': ab,ti

#7 'lung cancer': ab,ti

#8 'lung carcinoma': ab,ti

#9 'lung cancer'/exp

#10 OR/ (#6-9)

#11 molecular: ab,ti

#12 genomic: ab,ti

#13 'next-generation sequencing': ab,ti

#14 'protein expression': ab,ti

#15 OR/ (#11-14)

#16 #5 AND #10 AND #15
